# Translational Aspects of Cardiovascular Biology: From Bench to Bedside

**DOI:** 10.3390/biology12050658

**Published:** 2023-04-27

**Authors:** Gaetano Santulli

**Affiliations:** 1 Division of Cardiology, Department of Medicine, Wilf Family Cardiovascular Research Institute, Fleischer Institute for Diabetes and Metabolism (*FIDAM*), Einstein Institute for Neuroimmunology and Inflammation (*INI*), Albert Einstein College of Medicine, New York, NY 10461, USA; gsantulli001@gmail.com; 2 Department of Advanced Biomedical Sciences, International Translational Research and Medical Education (*ITME*) Consortium, “*Federico II*” University, 80131 Naples, Italy; 3 Department of Molecular Pharmacology, Einstein-Mount Sinai Diabetes Research Center (*ES-DRC*), Einstein Institute for Aging Research, Albert Einstein College of Medicine, New York, NY 10461, USA

Cardiovascular disease is the leading cause of death worldwide, and the search for novel mechanisms and therapeutics is desperately needed. Therefore, basic and translational studies in the cardiovascular field represent the best strategy to identify novel therapeutic targets, as well as to improve the quality of life of patients with cardiovascular disorders (Figure 1). This Editorial introduces the Special Issue “Translational Aspects of Cardiovascular Biology: From Bench to Bedside”, published in the Journal *Biology*. This Special Issue gather peer-reviewed scientific papers, which deal with cardiovascular medicine, from basic science to pre-clinical and clinical investigations, highlighting the crucial importance of translational medicine in testing groundbreaking hypotheses to advance the biomedical field.

The Special Issue opens with three basic research papers, which investigate endothelial cells. The first article shows that a specific non-coding RNA, microRNA-4432, is able to specifically target the gene encoding for fibroblast growth factor binding protein 1 (FGFBP1) in human brain microvascular endothelial cells, and this paper demonstrates that this microRNA significantly reduces endothelial oxidative stress, a well-established feature of hypertension [1]. The second study provides a demonstration of the extracellular metabolism of nicotinamide adenine dinucleotide (NAD+) and nicotinamide mononucleotide (NMN), multifunctional metabolites involved in a number of cellular processes, and which is vastly different in its expression in the vascular endothelium obtained from different species and locations [2]. The third paper, instead, deals with the functions and intracellular mechanisms of endothelial cells in human liver grafts [3].

Subsequently, a couple of in vitro investigations provide new insights on how several anticancer drugs, including Paclitaxel, Carboplatin, Doxorubicin, and Cyclophosphamide, can alter the biophysical characteristics of red blood cells [4]. These studies also examine why inhibiting isocitrate dehydrogenase 1 (IDH1) may help prevent foam cell formation by reducing oxidized low-density lipoprotein-induced ferroptosis in macrophages [5]. The following two papers harness two different animal models, mouse and swine, respectively, to show that dextran sodium sulfate-induced chronic colitis has no significant impact on femoral artery endothelial function or ischemic limb recovery [6]. Additionally, after myocardial infarction, neuregulin (NRG-1β) exhibits pro-myogenic and anti-cachectic actions in respiratory muscles [7]. In two elegant reviews, Bouhamida and colleagues explained the main effects of the interplay between hypoxia signaling on mitochondrial dysfunction and inflammation in cardiovascular diseases and cancer [8], while Nie and collaborators summarized the therapeutic potential of long non-coding RNAs (lncRNAs) in cardiac fibrosis [9].

The last section of the Special Issue includes a series of clinical studies, investigating tissue and serum biomarkers in degenerative aortic stenosis [10], identifying reliable biomarkers in obesity-related atrial fibrillation [11], establishing retinol-binding protein-4 as a predictor of insulin resistance in patients with coronary artery disease and type 2 diabetes mellitus [12], and verifying the correlation between carbonic anhydrase isozymes and the evolution of myocardial infarction in diabetic patients [13].

A double-blind, randomized, placebo-controlled clinical trial concludes the Special Issue, showing that vaccination with the pneumococcal vaccine *Prevenar-13* does not result in IgM against oxidized low-density lipoproteins, in contrast with previous findings in rodents [14].

## Figures and Tables

**Figure 1:**
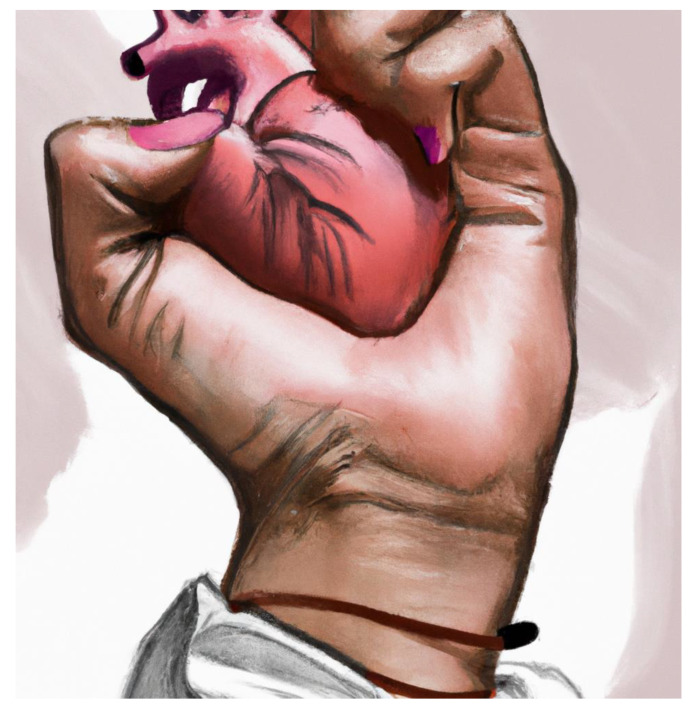
Artistic representation of the importance of translational medicine in the challenging quest for new therapies to cure cardiovascular disease.
